# The Influence of the Temperature on Effectiveness of Selected Disinfectants Against African Swine Fever Virus (ASFV)

**DOI:** 10.3390/v17020156

**Published:** 2025-01-24

**Authors:** Małgorzata Juszkiewicz, Marek Walczak, Grzegorz Woźniakowski, Zygmunt Pejsak, Katarzyna Podgórska

**Affiliations:** 1 Department of Swine Diseases, National Veterinary Research Institute, Partyzantów 57 Avenue, 24-100 Puławy, Poland; marek.walczak@piwet.pulawy.pl (M.W.); katarzyna.podgorska@piwet.pulawy.pl (K.P.); 2 Department of Infectious, Invasive Diseases and Veterinary Administration, Faculty of Biological and Veterinary Sciences, Nicolaus Copernicus University in Toruń, Lwowska 1 Street, 87-100 Toruń, Poland; grzegorz.wozniakowski@umk.pl; 3 University Veterinary Medicine Centre UJ-UR, University of Agriculture, al. Mickiewicza 21, 31-120 Kraków, Poland; zygmunt.pejsak@urk.edu.pl

**Keywords:** African swine fever virus, disinfection, biosecurity, sub-zero temperatures

## Abstract

African swine fever (ASF) is one of the most economically significant diseases of pigs caused by African swine fever virus (ASFV). Due to the lack of effective and safe vaccines, one of the crucial measures to protect farms from the introduction of the ASFV is to apply a strict regime of biosecurity and disinfection. However, in field conditions, the activity of disinfectants may be influenced by temperature, resulting in reduced activity or biodegradation (i.e., freezing or evaporating). The aim of this study was to evaluate the effect of a wide range of temperatures on the virucidal activity of selected active substances commonly used against ASFV. Eight active substances were tested, namely: sodium hypochlorite (1.0%), glutaraldehyde (0.1%), potassium peroxysulfate (0.5%), caustic soda (1.0%), phenol (1.0%), acetic acid (3.0%), benzalkonium chloride (1.0%), and formaldehyde (0.4%). The virucidal activity of each compound was tested at different temperatures (21, −10, and −20 °C for 30 min) and compared to the initial virus titer under the same temperature conditions. Exposure to a range of temperatures did not significantly affect the virucidal efficacy of tested active substances against ASFV. Most of the evaluated substances had reduced virus titers ≥ 4 log_10_, regardless of the temperature. However, two of them (benzalkonium chloride and acetic acid) were sensitive to sub-zero temperatures, showing a lack of the required 4 log_10_ virus titer reduction. The conducted study showed that temperature could hamper the virucidal effect of selected substances (i.e., benzalkonium chloride and acetic acid), showing their moderate efficacy against ASFV −10 °C and −20 °C. The results suggest that extreme caution should be taken while applying these substances at sub-zero temperatures. The other substances had no significant sensitivity to the temperature range. Nevertheless, in the case of freezing the agent, insufficient penetration of the disinfected surface may occur, which may result in an ineffective disinfection process.

## 1. Introduction

African swine fever (ASF) is one of the world’s most serious animal diseases caused by African swine fever virus (ASFV), causing huge losses in pig production and negatively impacting the economies of affected countries. The first occurrence of ASF and later introduction led to an endemic form of the disease in sub-Saharan Africa and in Sardinia, which in September 2024, after 40 years, was declared free of ASF by the European Commission [1,2]. The most recent epidemic began in Georgia in 2007 and resulted in the uncontrolled spread of the disease to Europe and other continents [3]. China, the world’s largest pork producer, has also experienced ASF outbreaks since 2018. From August 2018 to May 2020, China’s Ministry of Agriculture and Rural Affairs (MARA) confirmed 170 ASF outbreaks, resulting in 1.2 million pigs being stamped out, decimating Chinese pork production by over 40% [4,5]. A global decline in pork production of 12.9% could be observed between 2018 and 2020. Currently, China is gradually rebuilding its position in the global market, and by 2023, its share of the world’s pork production is expected to exceed 46% (55 million tons) [6]. Also, European countries faced a pig production crisis related to ASF. An example is Poland, one of the leading pig producers in Europe, struggling with ASF since 2014. According to experts’ opinions, Poland’s pig population in 2023 was 9 million—the lowest since 1945 [6,7]. One of the reasons for this decline is the spread of ASF, along with the European Commission tightening animal welfare legislation. The need to comply with increasingly costly breeding requirements has led some farmers to abandon pork production [7]. Poland has faced ASF since 2014, and has so far recorded more than 19,500 outbreaks in wild boars and 576 ASF outbreaks in swine (as of 05/11/2024) [8]. In total, ASF has been reported in 50 countries in five different regions of the world since January 2022. More than 484,000 pigs and more than 17,400 wild boars have been infected, resulting in more than 1,373,000 animal losses [9]. Presumably, the uncontrolled spread of the disease would be solved by the application of a vaccine, but despite years of work and extensive research, it has not been approved for use on the EU market. Only the Vietnamese government has approved the commercial use of two national ASF vaccines (i.e., NAVET-ASFVAC and AVAC ASF LIVE) [10]. Despite promising results, vaccination should be approached with caution, considering the events in Portugal and Spain during the 1960s, when a vaccine caused unpredicted post-vaccination side effects, including deaths, and created large numbers of carriers, complicating subsequent eradication efforts [11]. In the absence of a globally approved vaccine, and amid the global threat of ASF, WOAH emphasizes the importance of strict biosecurity measures, an early warning and response system, and heightened disease awareness among all those potentially at risk [9]. There are different regulations for approving disinfectants during notifiable disease outbreaks depending on the country, such as a quantitative carrier test where the virus is dried on a surface, or a quantitative suspension test.

ASF spreads through direct (natural hosts—wild boars and pigs) and indirect contact (i.e., contaminated vehicles or pork products). When considering measures to control the spread of ASF, these routes of transmission must be recognized, making biosecurity measures and disinfection essential tools for preventing the introduction of ASFV into pig farms. The effectiveness of disinfecting agents depends on variables such as sensitivity of targeted microbes, practical conditions, and the specific disinfectant being evaluated [12]. It can also be influenced by physical factors that affect the chemical reaction, including temperature, pH, disinfectant application, mechanical action, humidity, water hardness, and contact time. Low temperatures can preserve pathogens, freeze water, and make drying difficult, thereby triggering a significant limitation to vehicle disinfection [13]. The United States Department of Agriculture (USDA) recommends that when the ambient temperature is below freezing, either surfaces should be heated to prevent freezing, heat blankets should be used around liquid containers, or 40% propylene glycol in water could be added when mixing solutions [14].

Until now, several scientific articles have described the effect of temperature on the efficacy of disinfectants against viruses, bacteria, or fungi [11,12,13,14,15,16,17,18]. Several articles describing the sensitivity of ASFV to disinfectants have been published [15,16,17,18], using different tests based on country-specific regulations, such as the quantitative carrier test, in which the virus is dried on a surface, or a quantitative suspension test. However, the influence of the temperatures, especially freezing temperatures, on their effectiveness, has not been determined. Since disinfectants are also used outdoors (disinfection mats and basins) and are exposed to a wide range of temperatures, including temperatures below zero, their efficacy may be negatively affected. The present study was undertaken to test the effectiveness of selected chemicals used in the production of disinfectants against ASFV in a range of temperatures simulating both room temperature (21 °C) as well as winter scenarios (−10 °C and −20 °C) based on a quantitative suspension test. This test was evaluated, according to Krug et al.’s results, which resulted in ASFV not being very resistant to drying [19].

## 2. Materials and Methods

### 2.1. Cells and Viruses

The Vero-adapted Ba71V strain was obtained from the European Union Reference Laboratory, in Valdeolmos, Spain. ATCC Vero cell line (ATCC^®^ CCL-81^TM^) was subcultured in a Minimum Essential Medium (GIBCO, Life Technologies, Carlsbad, CA, USA), supplemented with 10% Fetal Bovine Serum (FBS) (Gibco, Billings, MT, USA) and a 1% Antibiotic Antimycotic Solution (100×) (Sigma-Aldrich, St. Louis, MO, USA). The cultures were incubated at 37 °C in a humidified atmosphere of air containing 5% CO_2_.

### 2.2. Virus Stock Preparation

Subconfluent monolayers of Vero cells were infected with 10^6^ TCID_50_/mL of the virus and were incubated at 37 °C until a 100% cytopathic effects were observed, usually after 4–5 days. In order to provide a sufficient virus titer (at least 10^5.5^ TCID_50_/mL), allowing for the demonstration of a 4 log_10_ reduction of titer after disinfectant treatment, the obtained viruses were subjected to 3 freeze/thaw cycles and precipitated using the following buffer: 20% Polyethylene glycol (PEG) and 2.5 M sodium chloride in a 2:3 buffer:virus ratio. The virus-buffer solution was agitated overnight at 4 °C, subsequently ASFV was pelleted by centrifugation at 13,000× *g* for 90 min at 4 °C and resuspended in 1/10 volume of the initial medium. The obtained virus stocks were titrated, aliquoted, and stored at −80 °C. Virus titers were determined by a 50% tissue culture infectious dose (TCID_50_/mL) titration, using the Spearman–Kärber method [20,21].

### 2.3. Disinfectants

The selected concentrations of each active substance were prepared immediately before use by dilution in hard water. The basic concentrations were selected, based on WOAH recommendation as being effective against ASFV and previous studies [17,22].

The following concentrations have been used, namely: formaldehyde (POCH, Gliwice, Poland, CAS: 50-00-0): 0.4%; sodium hypochlorite (solution in water containing 15% active chlorine, Stanlab, Lublin, Poland, CAS: 7681-52-9): 1%; caustic soda (POCH, Gliwice, Poland, CAS: 1310-73-2): 2%; glutaraldehyde (25%, Carl Roth, Karlsruhe, Germany, CAS: 111-30-8): 0.1%; phenol (Chempur, Piekary Śląskie, Poland, CAS: 108-95-2) in 1%; benzalkonium chloride (Pol-Aura, Olsztyn, Poland, CAS: 63449-41-2): 0.5%; potassium peroxymonosulfate (Envolab, Długomiłowice, Poland, CAS: 70693-62-8): 0.5%; acetic acid (Chempur, Piekary Śląskie, Poland, CAS: 64-19-7): 3%.

### 2.4. Diluent and Interfering Substances

All tested chemical compounds were diluted in water of standardized hardness, containing defined concentration of Mg^+^, Ca^2+^, Cl^−^, HCO^3−^ anions (pH 7). The hard water was prepared according to the PN-EN 14675:2015 European Standard [23]. The suspension test was prepared with interfering substances simulating low level soiling (BSA—bovine albumin 3.0 g/L) and high level soiling (BSA + YE—bovine albumin 10 g/L, plus yeast extract 10 g/L), according to the PN-EN 14675:2015 European Standard.

### 2.5. Temperature Conditions

Disinfectants at concentrations whose virucidal efficacy has been confirmed in previous studies [17] were incubated at 21 ± 1 °C, −10 ± 1 °C, and −20 ± 1 °C for 30 min in incubator and freezers with a continuously monitored temperature range, respectively.

### 2.6. Test Conditions

Each disinfectant was tested in triplicates. One part of the virus suspension was mixed with one part of the interfering substances, respectively, with BSA or BSA + YE and incubated at 21, −10, and −20 ± 1 °C for 2 min ± 10 s. Subsequently, eight parts of the disinfectant, diluted to 1.25-fold of each tested concentration, were added. The obtained mixture of virus, disinfectant and interfering substances was incubated at 21, −10 and −20 ± 1 °C for 30 min ± 10 s, afterwards the test tubes were placed on crushed ice (4 °C). The samples were immediately serially diluted (in quadruplicates) 10-fold (both the control virus and experimental virus suspensions) in a suspension of Vero cells, in 96-well plates. The plates were incubated for 7 days at 37 ± 2 °C in air containing 5% CO_2_ and examined daily for the appearance of cytopathic effects (CPEs). All the plates were ultimately scored for CPEs upon microscopic examination after 7 days. A minimum 5.5 log_10_ (TCID_50_/_mL_) of virus titers in the control sample was required to demonstrate a ≥4 log reduction. The study is based on the European Standard PN: EN 14675 which does not define the level of significant difference and does not require calculating it, instead focusing on a defined threshold value. Additionally for a disinfectant to be considered virucidal a reduction of titer of ≥4 log_10_ compared to the controls is needed.

### 2.7. Cytotoxicity Reduction

**Figure 1:**
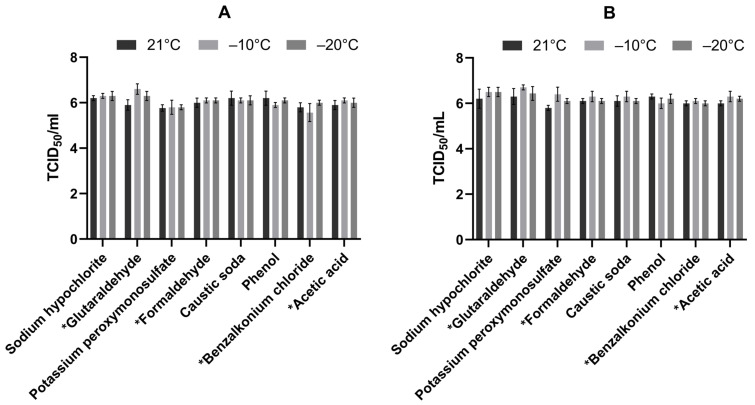
Mean titer of the virus control in the presence of (**A**) low soiling level (BSA) and (**B**) high soiling level (BSA + YE) in three different temperature conditions (21 °C, −10 °C, and −20 °C). The medium detectable log_10_ of the virus control, considering the standard deviation, are presented. *—mean titer of the virus control with micro-filtration.

Several chemical agents turned out to be cytotoxic to the Vero cells, therefore precluding in proper test performance and the demonstration of a 4 log_10_ titre reduction. Microspin S-400 HR columns (GE Healthcare, Fairfield, CT, USA) were used to remove the cytotoxic agent from the samples, right after 30 min of incubation of the tested and control samples. Virus controls with and without micro-filtration were included to estimate the loss in the virus titer (Figure 1).

### 2.8. Test Controls

In control samples, the chemicals were replaced by hard water. Both standard and cytotoxicity controls were processed in the same manner as the chemical compounds tested. The mean titer of the virus control was show in Figure 1.

### 2.9. Statistical Analysis

Statistical analyses were performed using the GraphPad Prism (version 8.4.3, La Jolla, CA, USA). Analyses of the mean differences between each disinfectant were shown with a standard deviation.

## 3. Results

To determine the efficacy of the chemical compounds at different temperatures, the mean log of the virus treated with the chemical compound was compared to the titer of the control virus. A disinfectant was considered virucidal if a reduction of ≥4 log_10_ compared with the control was demonstrated. The results of the initial in vitro suspension tests of chemical compounds are summarized in the Table 1.

**Figure 2:**
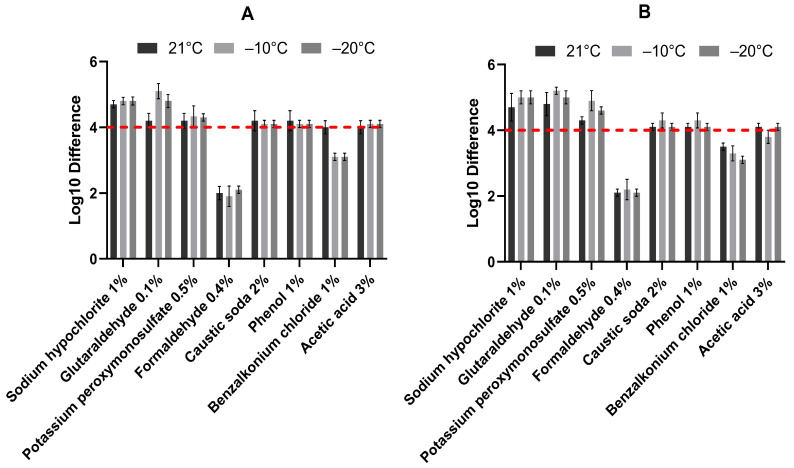
The influence of the temperature on the effectiveness of selected disinfectants, in the presence of (**A**) low soiling level (BSA) and (**B**) high soiling level (BSA + YE), in three different temperature conditions (21, −10, and −20 °C). The medium detectable log_10_ differences between the control and tested samples, considering the standard deviation, are presented. The red dashed line—virucidal effect threshold.

The influence of temperature (21, −10, and −20 °C) on the efficacy of eight active substances used in the production of commercial disinfectants against ASF was evaluated by a modified method of European Standard EN 14675: 2015. A noticeably lower logarithmic reduction was observed in the cases of glutaraldehyde (BSA) and potassium peroxymonosulfate (BSA + YE) incubated at 21 °C. Low temperatures had no significant impact on the efficacy of most of the chemical compounds tested, except for benzalkonium chloride, for which it reduced its virucidal activity, as shown in Figure 2.

It is worth mentioning that sub-zero temperatures led to the freezing of each of the tested chemicals, which has a key impact on the penetration properties of the disinfectant and may result in ineffective disinfection.

The virucidal efficacy within the tested temperature range (21, −10, and −20 °C) was confirmed for most of the evaluated active substances. Three active substances tested: sodium hypochlorite, glutaraldehyde and potassium peroxymonosulphate, were the most effective, reducing the ASFV titer by approximately 5 log_10_ despite temperature conditions. As can be seen in Table 1, potassium peroxymonosulphate performed better in inactivating the virus at sub-zero temperatures under high level soiling conditions, achieving 4.6 (±0.11) and 4.9 (±0.31) log_10_ reductions at temperatures of −10 and −20 °C, respectively, compared to the results obtained at 21 °C. Whereas the same substance under low soiling conditions appeared more stable to varying temperature conditions, reducing the initial virus titer to a similar level of 4.2–4.3 log_10_. A similar effect was observed for glutaraldehyde (BSA), which showed decreased virucidal efficacy at room temperature compared to sub-zero temperatures. Sodium hypochlorite showed to be the most stable to temperature changes and the presence of organic material chemical compounds. The results at sub-zero temperatures, depending on the soiling conditions, were 4.8 and 5.0 log_10_ reduction, while at room temperature they were 4.7, irrespective of the level of organic matter. Caustic soda, phenol, and acetic acid showed virucidal efficacy against ASFV at similar levels of reduction (Figure 2). Two of these were effective at tested temperature ranges in both soiling conditions, except acetic acid which showed to be sensitive to the −10 °C temperature (BSA + YE) and reduced the initial virus titer by 3.8 (±0.2) log_10_. The lowest efficacy was observed for benzalkonium chloride, which belongs to a quaternary ammonium compound (QAC). The benzalkonium chloride showed efficacy bordering on the required level (4.0 (±0.2) log_10_ titre reduction) only at room temperature (21 °C) under low soiling conditions. Sub-zero temperatures in combination with high level soiling conditions had a negative effect on the agents’ activity, which indicates its sensitivity to those conditions.

Samples showing high cytotoxicity in previous studies [17] (formaldehyde, glutaraldehyde, benzalkonium chloride, and acetic acid) were additionally subjected to Microspin S-400 HR filtration. Microfiltration resulted in ≤0.3 log_10_ loss of the initial virus titer, however, cytotoxicity was reduced from 3 to ≥1 log_10_, therefore allowing for the assessment of the effectiveness of the three compounds. Microfiltration was insufficient to reduce the cytotoxicity of formaldehyde, which showed the highest cytotoxicity in the initial testing. The highest reduction in virus titer achieved by formaldehyde was about 2 log_10_.

## 4. Discussion

ASF is the most serious disease of pigs and wild boars, and it has a huge impact on the economies of the affected countries. The growing occurrence of the disease is illustrated by the number of countries that have faced ASF epizootic. As of 12 July 2024, 74 countries have registered ASF on their territories [24]. Biosecurity, combined with effective disinfection, plays a key role in limiting the spread of the disease. Therefore, when selecting a proper disinfectant, a range of factors should be considered, including effectiveness against the specific pathogen(s), effectiveness in the presence of organic matter, and over a wide temperature range. The temperature is considered to be one of the most important environmental conditions that can affect the virucidal efficacy of disinfectants. To our knowledge, this is the first in vitro suspension study addressing the virucidal activity of chemical compounds against ASFV exposed to a wide range of temperatures, based on a modified PN-EN 14675:2015 European Standard.

Seven out of the eight tested chemicals (e.g., sodium hypochlorite, glutaraldehyde, potassium peroxymonosulfate, caustic soda, phenol, acetic acid, and benzalkonium chloride) caused effective ASFV inactivation at 21 °C. Similarly to previous studies [15] (temperature 10 °C), benzalkonium chloride, which belongs to a quaternary ammonium compound (QAC), showed the lowest efficacy. Low temperatures had a negative effect on the efficacy of this agent. Previous studies by Stefanello et al. and Aksoy et al. have found that the increase in biocidal and antifungal efficacy of benzalkonium chloride is directly proportional to the increase in temperature, which is in line with our findings [25,26]. In addition, Jang et al. showed that low temperatures (−10 °C) inhibited the virucidal efficacy of QAC on enveloped viruses [27]. High soiling conditions had a negative impact on the agents’ activity, even at room temperature, which indicates the importance of using detergents before proper disinfection. The organic matter can hamper the effectiveness of disinfectants but also can lead to better survival of pathogens, as presented by Krug et al., who observed the effect of higher serum concentrations on survival and viral proliferation [28]. Consistent with previous studies [17], formaldehyde remained cytotoxic despite microfiltration. Incubation under varying temperature conditions had no effect on the cytotoxic properties of formaldehyde, therefore its virucidal activity against ASFV could not be defined.

As shown by standard deviation and depending on the scenario, acetic acid could not sufficiently inactivate ASFV at −10 °C in high level soiling conditions. Consistent observations on the efficacy of acetic acid on enveloped viruses at temperatures of −10 °C were discussed by Jang et al., where lower temperatures reduced the effectiveness of citric acid [27]. Obviously, it should be considered that these are two different acids, however, the principles of their action were the same [29]. The effect of negative temperatures on peracetic acid and potassium bisulfate was studied by Ren et al. The authors proved the efficacy of the evaluated compounds at −20 °C on poliovirus (a non-enveloped virus) [30]. The results are consistent with the present study, where the efficacy of potassium peroxymonosulphate at all temperatures tested was confirmed. The high virucidal efficacy of caustic soda was already described in our previous studies [17]. Our present research has conclusively confirmed the effectiveness of caustic soda at sub-zero temperatures (−10 °C and −20 °C). However, caution should be taken when using caustic soda due to its exothermic properties, which may be augmented with a decreasing temperature. Mixing the chemical with water may lead to an explosive reaction, in which heat and hydrogen are released [31].

Sodium hypochlorite, glutaraldehyde, and potassium peroxymonosulphate were the most effective, reducing the ASFV titer by approximately 5 log_10_, and showing a comparable or better virucidal effect at sub-zero temperatures under high level soiling conditions. A similar effect was observed for glutaraldehyde, which under low soiling conditions showed lower virucidal efficacy at room temperature (21 °C) compared to sub-zero temperatures. The antimicrobial efficacy of glutaraldehyde at low temperatures has been previously reported [32]. Similarly to studies by Knud et al. it was shown that glutaraldehyde stored at −14 °C retained its properties for up to four months longer than that stored at 4 °C, confirming our study in which glutaraldehyde incubated at lower temperatures achieved slightly higher values for virus titer reduction [33]. The degradation effect of high temperatures (60 °C) on glutaraldehyde was described by McGinley et al. [34].

Sodium hypochlorite showed the highest efficiency under both low and high soiling conditions over the full spectrum of tested temperatures, therefore it can be considered the most resistant to the presence of organic matter and a wide range of temperatures. Our conclusions support previous studies, which confirmed the high resistance of this agent to a range of physical conditions, including drying [19], temperature [35,36], and the presence of organic matter [28].

Although each of these compounds has an apparently different mechanism of action, both sodium hypochlorite and potassium peroxymonosulphate are highly oxidative disinfectants and are effective in breaking down the integrity of cell membranes or viral capsids, inactivating the nucleic acids of viruses and causing metabolic disruption [37,38,39,40]. Despite its lack of oxidative capacity, glutaraldehyde, like the two agents mentioned above, penetrates bacterial cell walls and the outer layers of viruses by denaturing proteins, leading to disruption of the metabolism of protein-DNA cross-links and capsid alterations, resulting in increased permeability and eventual leakage of contents [41,42]. However, it should be remembered that although glutaraldehyde, sodium hypochlorite and potassium peroxymonosulphate are highly effective, they should be used with caution as they can be highly irritating, causing respiratory problems, skin irritation and allergic reactions, and may even be carcinogenic with prolonged use [42,43].

In summary, our present studies proved that sodium hypochlorite, glutaraldehyde, potassium peroxymonosulphate, caustic soda, and phenol showed efficacy at the given temperatures (21, −10, and −20 °C). The exception were benzalkonium chloride and acetic acid, which have shown sub-zero temperatures sensitivity and moderate efficacy against ASFV, suggesting that specific precautions should be taken into account when using these substances at negative temperatures. The highest virucidal activity against ASFV in temperatures tested has been shown in the cases of sodium hypochlorite, glutaraldehyde and potassium peroxymonosulfate. Our previous studies of chemical compounds showed high cytotoxity of QUATs and formaldehyde, which concur with present results assessment [17]. The high cytotoxicity of formaldehyde prevented a proper evaluation of its virucidal properties against ASFV. Therefore, in this study, we confirmed the low virucidal activity of QUATs represented by: benzalkonium chloride which is due as cytotoxicity affected the maximum reportable titer reductions and which has recently been reported [17]. Acetic acid proved to be sensitive to −10 °C temperatures. The resulting reduction in the initial virus titer did not reach the required 4 log, which brings into doubt its efficacy against ASFV. It should be mentioned that all the evaluated agents got frozen during incubation at sub-zero temperatures.

## 5. Conclusions

Although virucidal activity was maintained for most of the chemicals despite freezing, their activity towards virus suspension may not reflect the situation in field conditions may be influenced by additional factors that we were not able to take into account in the laboratory tests. Freezing a disinfectant may significantly impact its physical properties (e.g., viscosity) and cause limited penetration throughout disinfected surfaces. Any divergence from the optimal temperature defined for a given disinfectant may limit its effectiveness due to increased evaporation or freezing, which can lead to ineffective disinfection. It is important to consider that the principles of proper biosecurity apply in all seasons and in all weather conditions.

Overall, the results of the present study indicated the need for future research focused on identifying ingredients that would prevent disinfectants from freezing, as this may improve penetration and potentially enhance biocidal effectiveness.

## Figures and Tables

**Table 1:** Logarithmic reduction of ASFV titers in the presence of tested active substances. Contact time, 30 min. Temperature of incubation, 21, −10, −20 °C. Each data point is the mean of three experiments, ±SD.

Active Substance	Temperature (C)	Log_10_ Difference ** (±SD) (TCID_50_/mL)		Virucidal Effect (Difference ≥ 4 Log_10_)	
		BSA	BSA + YE	BSA	BSA + YE
Sodium hypochlorite (1%)	21	4.7 (±0.11)	4.7 (±0.42)	Yes	Yes
	−10	4.8 (±0.11)	5.0 (±0.2)	Yes	Yes
	−20	4.8 (±0.12)	5.0 (±0.2)	Yes	Yes
Glutaraldehyde (0.1%) *	21	4.2 (±0.23)	4.8 (±0.35)	Yes	Yes
	−10	5.1 (±0.23)	5.2 (±0.11)	Yes	Yes
	−20	4.8 (±0.2)	5.0 (±0.2)	Yes	Yes
Potassium peroxymonosulfate (0.5%)	21	4.2 (±0.23)	4.3 (±0.11)	Yes	Yes
	−10	4.3 (±0.31)	4.9 (±0.31)	Yes	Yes
	−20	4.3 (±0.11)	4.6 (±0.11)	Yes	Yes
Formaldehyde (0.4%) *	21	2.0 (±0.2)	2.1 (±0.11)	N/A	N/A
	−10	1.9 (±0.31)	2.2 (±0.31)	N/A	N/A
	−20	2.1 (±0.11)	2.1 (±0.11)	N/A	N/A
Caustic soda (2%)	21	4.2 (±0.31)	4.1 (±0.11)	Yes	Yes
	−10	4.1 (±0.11)	4.3 (±0.23)	Yes	Yes
	−20	4.1 (±0.11)	4.1 (±0.11)	Yes	Yes
Phenol (1%)	21	4.2 (±0.31)	4.1 (±0.11)	Yes	Yes
	−10	4.1 (±0.11)	4.3 (±0.23)	Yes	Yes
	−20	4.1 (±0.11)	4.1 (±011)	Yes	Yes
Benzalkonium chloride (1%) *	21	4.0 (±0.2)	3.5 (±0.11)	Yes	No
	−10	3.1 (±0.11)	3.3 (±0.23)	N/A	N/A
	−20	3.1 (±0.11)	3.1 (±011)	N/A	N/A
Acetic acid (3%) *	21	4.0 (±0.2)	4.1 (±0.11)	Yes	Yes
	−10	4.1 (±0.11)	3.8 (±0.2)	Yes	Yes
	−20	4.1 (±0.11)	4.1 (±0.11)	Yes	Yes

**—Difference between control and tested sample, *—Cytotoxic effect, N/A—virucidal effect could not be evaluated due to cytotoxicity. BSA—low soiling level (bovine serum albumin 3.0 g/L), BSA + YE—high soiling level (bovine serum albumin 10.0 g/L + yeast extract 10.0 g/L).

## Data Availability

The raw data supporting the conclusions of this article will be made available by the authors on request.
